# Multicenter evaluation of ceftazidime-avibactam use in carbapenem-resistant *Klebsiella pneumoniae* bloodstream infections in OXA-48 endemic regions

**DOI:** 10.1038/s41598-024-77259-z

**Published:** 2024-11-01

**Authors:** Ali Mert, Okan Derin, Halis Akalın, Rıdvan Dumlu, Sibel Gündeş, Rehile Zengin, Sesin Kocagöz, Yasemin Gündoğdu, İftihar Köksal, Demet Yalçın, Cemal Üstün, Mahir Kapmaz, Levent Görenek, Kadriye Karahangil, Füsun Can, Consortium, Uğur Önal, Süda Tekin, Rıza Aytaç Çetinkaya, Didem Akal Taşçıoğlu, Gülay İmadoğlu Yetkin, Sedef Başgönül, Serap Gençer, Ece Akbulut, Ferhat Arslan, Güneş Şenol, Cenk Kıraklı, Şirin Menekşe, Servet Alan, Nevin Sarıgüzel, Elif Hakko, Mehmet Karabay, Funda Şimşek, Sibel İba Yılmaz, Esin Çevik, Bülent Kaya, Önder Ergönül

**Affiliations:** 1 Department of Internal Medicine, İstanbul Medipol University, Faculty of Medicine, Istanbul, Turkey; 2 Department of Infectious Diseases and Clinical Microbiology, Istanbul Şişli Hamidiye Etfal Training and Research Hospital, Istanbul, Turkey; 3 Epidemiology PhD Program, Graduate School of Health Sciences, Istanbul Medipol University, Istanbul, Turkey; 4 Department of Infectious Diseases and Clinical Microbiology, Bursa Uludağ University, Faculty of Medicine, Bursa, Turkey; 5 Department of Infectious Diseases and Clinical Microbiology, Istanbul Medipol University, Faculty of Medicine, Istanbul, Turkey; 6 Department of Infectious Diseases and Clinical Microbiology, Bahcelievler Memorial Hospital, Istanbul, Turkey; 7 Department of Infectious Diseases and Clinical Microbiology, Acıbadem Altunizade Hospital, Istanbul, Turkey; 8 Department of Infectious Diseases and Clinical Microbiology, Acıbadem University, Faculty of Medicine, Istanbul, Turkey; 9 Department of Internal Medicine, Acıbadem Atakent Hospital, Istanbul, Turkey; 10 Department of Internal Medicine, Acıbadem University, Faculty of Medicine, Istanbul, Turkey; 11 Department of Infectious Diseases and Clinical Microbiology, Acıbadem Atakent Hospital, Istanbul, Turkey; 12 Department of Infectious Diseases and Clinical Microbiology, Bahçeşehir University, Faculty of Medicine, Göztepe Medical Park Hospital, Istanbul, Turkey; 13 Department of Infectious Diseases and Clinical Microbiology, Acıbadem Bakırköy Hospital, Istanbul, Turkey; 14 Department of Infectious Diseases and Clinical Microbiology, Koç University, Faculty of Medicine, Istanbul, Turkey; 15 Department of Infectious Diseases and Clinical Microbiology, University of Health Sciences, Hamidiye Faculty of Medicine, Istanbul, Turkey; 16 Department of Infectious Diseases and Clinical Microbiology, Bagcilar Medicine Hospital, Istanbul, Turkey; 17 Department of Medical Microbiology, Koç University, Faculty of Medicine, Istanbul, Turkey; 18 Department of Infectious Diseases and Clinical Microbiology, İstinye University, Faculty of Medicine, Bahçeşehir Liv Hospital, Istanbul, Turkey; 19 Department of Medical Microbiology, İstinye University, Faculty of Medicine, Bahçeşehir Liv Hospital, Istanbul, Turkey; 20 Department of Infectious Diseases and Clinical Microbiology, Acıbadem Maslak Hospital, Istanbul, Turkey; 21 Department of Infectious Diseases and Clinical Microbiology, Medeniyet University, Faculty of Medicine, Göztepe Hospital, Istanbul, Turkey; 22 Department of Infectious Diseases and Clinical Microbiology, University of Health Sciences, İzmir Faculty of Health Sciences, Suat Seren Training and Research Hospital, İzmir, Turkey; 23 Department of Infectious Diseases and Clinical Microbiology, Koşuyolu High Specialty Education and Research Hospital, Istanbul, Turkey; 24 Department of Infectious Diseases and Clinical Microbiology, Şişli Memorial Hospital, Istanbul, Turkey; 25 Department of Infectious Diseases and Clinical Microbiology, Acıbadem Kozyatağı Hospital, Istanbul, Turkey; 26 Department of Infectious Diseases and Clinical Microbiology, Anadolu Medical Center, Istanbul, Turkey; 27 Department of Infectious Diseases and Clinical Microbiology, Bahçelievler Medicana Hospital, Istanbul, Turkey; 28 Department of Infectious Diseases and Clinical Microbiology, University of Health Sciences, Hamidiye Faculty of Health Sciences, Prof. Dr. Cemil Taşçıoğlu City Hospital, Istanbul, Turkey; 29 Department of Infectious Diseases and Clinical Microbiology, Erzurum Regional Education and Research Hospital, Erzurum, Turkey; 30 Department of Infectious Diseases and Clinical Microbiology, Demiroğlu Bilim University, Faculty of Medicine, İstanbul Florence Nightingale Hospital, Istanbul, Turkey; 31 Department of Infectious Diseases and Clinical Microbiology, Kartal Dr. Lütfi Kırdar City Hospital, Istanbul, Turkey

**Keywords:** Bloodstream infections, Carbapenem-resistant Klebsiella pneumoniae, Ceftazidime-Avibactam, OXA-48, OXA-48 endemic area, Bacterial infection, Antimicrobial resistance

## Abstract

Data in the literature on the use of ceftazidime-avibactam (CAZ-AVI) in carbapenem-resistant *Klebsiella pneumoniae* bloodstream infections (CRKP-BSIs) are limited especially in OXA-48 (Oxacillinase-48) predominant regions. Our study aimed to evaluate the effect of CAZ-AVI use on outcomes in CRKP-BSIs in Turkey, where OXA-48 is endemic. A multicenter retrospective observational study was conducted between January 2017 and September 2021. The effects of clinical and treatment characteristics on 30-day mortality and relapse in CRKP-BSIs were analyzed. Predictors of outcomes were detected using a Cox regression model. The study enrolled 106 adults with CAZ-AVI-sensitive CRKP-BSIs who received CAZ-AVI for at least 72 h. Patients who received CAZ-AVI as initial therapy had lower mortality rates when compared to those who switched from last resort regimens [14.3% (*n* = 3/21) vs. 37.7% (*n* = 32/85), *p* = 0.04]. In multivariate analysis, older age and severe neutropenia were detected to be associated with higher mortality, significantly. Initiation of CAZ-AVI on the day of blood culture was obtained, was found to be significantly associated with lower mortality (HR: 0.25, CI: 0.07–0.84, *p* = 0.025). CAZ-AVI monotherapy is an important treatment option for CRKP-BSIs in OXA-48 endemic areas. Early initiation of CAZ-AVI should be preferred rather than switching from a last-resort regimen as it profoundly improves the survival rates.

## Introduction

Carbapenem-resistant *Klebsiella pneumoniae* (CRKP) infection is a global public health problem affecting Turkey and many other countries^1^. The first OXA-48 (Oxacillinase-48)-producing K. pneumoniae isolate was identified in Turkey in 2001^2^ and since its identification, it has become endemic in Turkey and has spread to many regions, especially Europe, the Middle East and the North Africa^1–3^.

Avibactam, a novel non-β-lactam β-lactamase inhibitor, inhibits OXA-48 carbapenemases. Therefore, ceftazidime-avibactam (CAZ-AVI) can be used to treat OXA-48-producing *K. pneumoniae* bloodstream infections (BSIs) in adult patients with limited treatment options^4^. There is limited experience with CAZ-AVI’s indication in CRKP bloodstream infections (BSIs). Therefore, we aimed to present the factors affecting the outcome in CRKP-BSIs patients treated with CAZ-AVI in Turkey, a population where OXA-48-producing *K. pneumoniae* is endemic.

## Materials and methods

### Study population and setting

We conducted a multicenter, retrospective, observational study in 23 hospitals in Turkey, including patients with CRKP-BSIs treated with CAZ-AVI between January 1, 2017, and September 31, 2021. The data were collected via Microsoft Forms between November 1, 2021, and December 31, 2021. Patients’ age, gender, Pittsburg Bacteremia Index score, Charlson Comorbidity Index score, presence of neutropenia, presence of malignancy, antibiotic susceptibility test results, and the duration between the blood culture collection and the initiation of antibiotic treatment, duration of treatment, 30-day mortality and recurrence rate after treatment were recorded. The effect of the patients’ demographic, clinical, and treatment characteristics on the outcomes were analyzed.

### Study approval

The study received approval from the Medipol University Ethics Committee (E-10840098-772.02-2738) and complied with the ethical principles of the Declaration of Helsinki for medical research involving human subjects.

### Inclusion criteria

We included adult patients (≥ 18 years),

Patients whose blood culture-confirmed monomicrobial CAZ-AVI-susceptible CRKP-BSIs, Patients who received CAZ-AVI monotherapy within the first 7 days of blood culture positivity.

### Exclusion criteria

Patients who received CAZ-AVI treatment for less than 72 h,

Patients treated with CAZ-AVI in combination therapy,

Patients with incomplete or inaccessible data were excluded from the study.

### Microbiological analysis

Blood culture samples were processed using the BacT/ALERT 3D system (bioMérieux, France). Positive growth signals were assessed by Gram staining and cultured on sheep blood agar, MacConkey agar, and chocolate agar (all bioMérieux, France). Plates were incubated for 24–48 h at 37 °C. Isolates were identified by the Matrix-Assisted Laser Desorption/Ionization-Time of Flight (MALDI-TOF MS) system (bioMérieux, France). In vitro, antibiotic susceptibility was determined using the VITEK^®^ 2 system (bioMérieux, France) or Phonenix^®^ system (Becton Dickinson, USA), according to each center’s protocols. CAZ-AVI susceptibility was determined using the Kirby-Bauer disc diffusion method. Susceptibility thresholds were ≥ 13 mm for CAZ-AVI according to the criteria of the European Committee on Antimicrobial Susceptibility Testing (EUCAST)^5^.

### Treatment protocol

CAZ-AVI was administered intravenously at a standard dose of 2/0.5 g every 8 h as a 2-hour infusion. The dose was adjusted for patients with renal failure according to the manufacturer’s instructions^6^. CAZ-AVI was used either as the initial treatment or as salvage therapy during the infection course.

### Outcome definitions

Mortality cases were defined as those in which death occurred within the first 30 days following the initiation of treatment. Relapse is defined as the recurrence of infection within 30 days after the end of treatment. Clinical success was defined as survival and the absence of recurrence 30 days after the onset of infection.

### Statistical analysis

Categorical and continuous variables were compared by using chi-square and Mann–Whitney U tests, respectively. The statistical significance was set as “p” value of < 0.05 (2-sided test). Non-normal data were analyzed by using Mann-Whithey U test. In multivariate analysis for the prediction of the fatality, gender, age, Charlson comorbidity index, severe neutropenia (neutrophil count < 500), starting CAZ-AVI on the day of blood culture was obtained were included to the model. The cox regression model was performed, hazard ratio (HR) with 95% confidence interval was calculated, and the Kaplan-Meier curve was constructed.

## Results

We included 106 adults with CAZ-AVI susceptible CRKP-BSIs who received at least 72 h of CAZ-AVI treatment. Demographic characteristics and univariate analysis of 106 patients with CRKP bacteremia who received the CAZ-AVI within 7 days of positive blood culture were presented in Table 1. Mean age was higher in fatal cases than the survived ones (59 vs. 51, *p* = 0.033). Mean Pittsburg bacteremia score was significantly higher among the fatal cases than the survived ones (7 vs. 4.1, *p* < 0.001), however; no significant difference between fatal and survived cases was detected in Charlson comorbidity index, having malignancy or having neutropenia (Table 1).

**Table 1:** Univariate analysis of 106 patients with Carbapenem-Resistant-*Klebsiella pneumonia* bacteremia who received the ceftazidime-avibactam (CAZ-AVI) within 7 days of positive blood culture.

	Survived *n* = 71 (67%)	Fatal *n* = 35 (33%)	*p*
Male gender	44 (62)	21 (60)	0.845
Mean Age	51 (sd: 17)	59 (sd: 18)	0.033
Mean Pitt bacteremia score	4.1 (sd: 3.2)	7 (sd: 2.6)	< 0.001
Charlson Comorbidity index	5.2 (sd: 11.6)	4.3 (sd: 2.7)	0.648
Severe neutropenia	12 (17)	9 (26)	0.284
Malignancy	27 (38)	14 (40)	0.845
Mean initiation time of CAZ-AVI after blood culture collection (days)	2.1 (sd: 1.9)	2.9 (sd: 1.85)	0.035
Patients initiated with CAZ-AVI on the day of blood culture was obtained	23 (32.3)	3 (8.5)	0.007

The mean initiation time of CAZ-AVI after blood culture collection was 2.1 days among the survived cases, whereas it was 2.9 days among the fatal cases (*p* = 0.035). Initiating CAZ-AVI on the day of blood culture was obtained was significantly more common among the survived cases than the fatal cases (32.3% vs. 8.5%, *p* = 0.007) (Table 1). Mortality was found to be lower in those who started CAZ-AVI as first-line treatment compared to those who switched treatment to CAZ-AVI [14.3% (*n* = 3/21) vs. 37.7% (*n* = 32/85) (chi-square test *p* = 0.04, and fisher exact test *p* = 0.06)]. No development of resistance or relapse was observed in any of the cases.

**Fig. 1:**
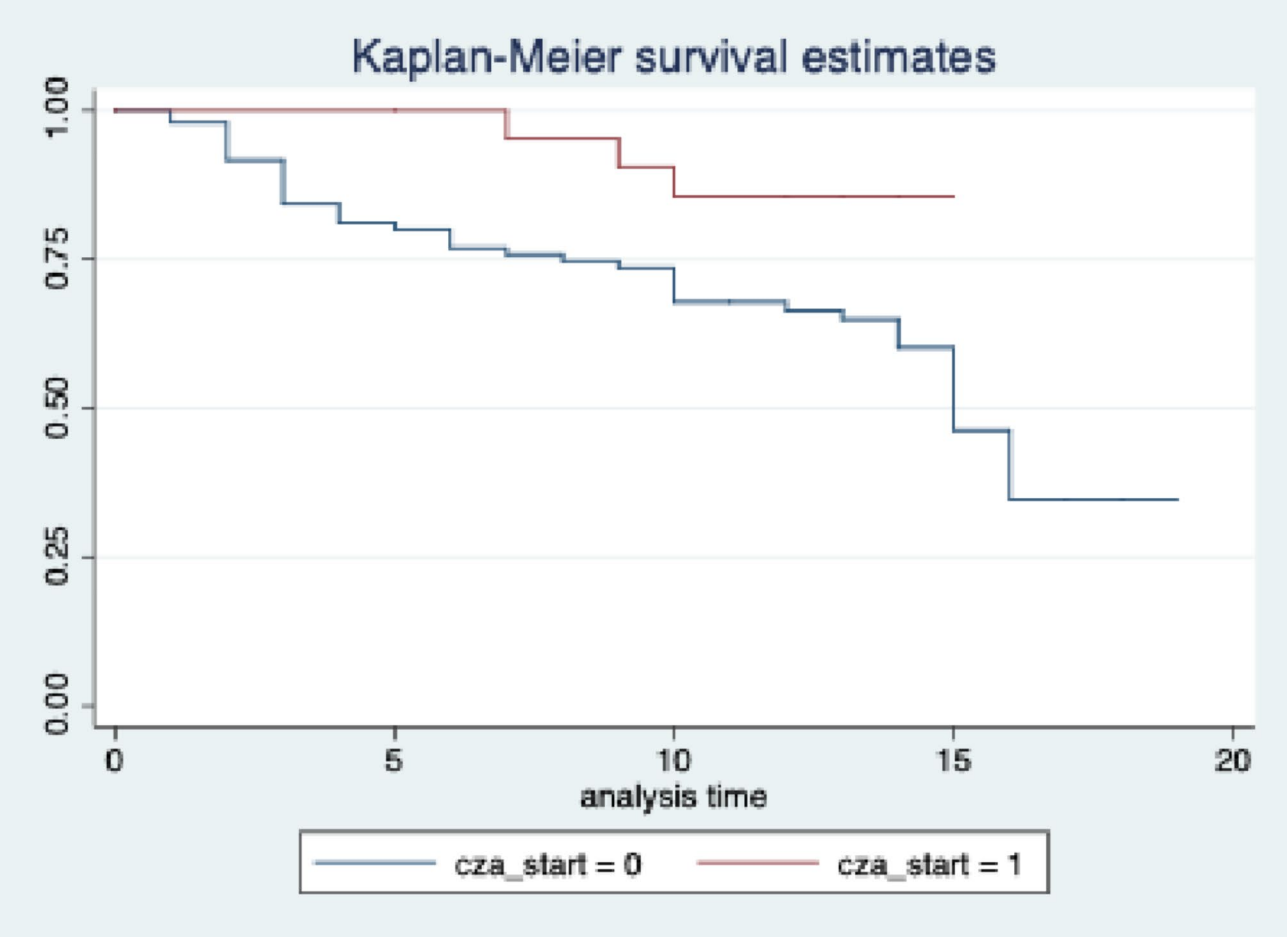
The role of ceftazidim-avibactam (CAZ-AVI) initiated on the day of blood culture was obtained compared to CAZ-AVI started later days in predicting 30-day fatality.

In multivariate analysis for the prediction of the fatality, gender, age, Charlson comorbidity index, severe neutropenia (neutrophil count < 500), initiating CAZ-AVI on the day of blood culture was obtained were included in the model (Table 2). There was a significant association between older age, having severe neutropenia and significantly higher fatality rates. Furthermore, the study projected that initiating CAZ-AVI on the day of blood culture was obtained compared to switching treatment from colistin-based-regimen to CAZ-AVI significantly reduced fatality (HR:0.25, CI: 0.07–0.84, *p* = 0.025) (Table 2; Fig. 1).

**Table 2:** Univariate and multivariate analysis (cox regression) for the predictors of fatality among the patients with Carbapenem-resistant Klebsiella pneumonia blood stream infection (BSI) who received ceftazidime-avibactam (CAZ-AVI) within 7 days after bacterial identification (*n* = 106 patients with CRKP-BSI, who received CAZ-AVI).

	Univariate			Multivariate		
	HR	CI	P	HR	CI	p
Male gender	0.89	0.45–1.75	0.743	0.91	0.45–1.81	0.796
Age	1.02	1.01–1.04	0.033	1.04	1.01–1.07	0.004
Charlson comorbidity index	0.99	0.94–1.03	0.771	0.96	0.83–1.11	0.599
Severe Neutropenia (neutrophil count < 500)	1.54	0.72–3.29	0.264	4.4	1.60-12.56	0.004
Patients initiated with CAZ-AVI on the day of blood culture was obtained	0.22	0.07–0.74	0.015	0.24	0.07–0.79	0.019

## Discussion

The OXA-48-producing *K. pneumoniae* is endemic in Turkey. CRKP-BSIs are associated with high mortality as treatment options are limited. Data from clinical trials on the management of CRKP-BSIs is scarce^7–10^. The data from observational studies recommend the use of ‘last-resort’ agents (e.g., colistin, polymyxin-B, aminoglycosides, fosfomycin, tigecycline, and/or carbapenems) only as a combined therapy when there is no access to CAZ-AVI and the bacteria is susceptible to the aforementioned agents^11–13^. The studies show that the 30-day mortality rate in polymyxin-based last-resort regimens for KPC or OXA-48-producing CRKP-BSIs is around 50%^11–13^. CAZ-AVI treatment in CRKP-BSIs was reported to have lower mortality rates than the ‘last-resort’ antibiotic regimens (~ 25% vs. ~ 50%)^6–12^. In our study, the 30-day mortality rate of the patients treated with CAZ-AVI was 33%.

Additionally, a recent multicentered study from Turkey reports that OXA-48-like carbapenemases comprised the largest carbapenemase group with 75% of CRKP bloodstream isolates, and 16% of these isolates were accompanied with NDM type beta-lactamases^14^. In other study in Turkey^15^, OXA-48 and NDM-1 positivity rates were found to be 81% and 19%, respectively, among carbapenem- and colistin-resistant *K. pneumoniae*.

We detected that initiating CAZ-AVI on the day of blood culture was obtained significantly improved the survival compared to delayed initiation of treatment. Our results were consistent with the findings of Falcone et al. reported that shortening the median time to a targeted antibiotic therapy improved survival in CRKP-BSIs^16^.

Resistance may develop during treatment with CAZ-AVI. In various studies, it is reported that 1.4–3.6% of CRKP-BSIs patients develop resistance after administration of CAZ-AVI^8–10^. In our cohort, no resistance was observed during CAZ-AVI treatment.

In Turkey; a study showed high CAZ-AVI susceptibility rates: 92% in *K. pneumoniae*, 91% in all Enterobacterales, and 82% in carbapenem-resistant Enterobacterales (CRE) isolates^17^. Another study also reported that the prevalence of resistance to the ‘last-resort’ antibiotics in CRKP has been increasing^15^. As a result of the increasing prevalence of resistance to the last resort antibiotics, CAZ-AVI emerges as a first-line therapy for CRKP infections.

The main risk factors predicting the mortality in patients of CRKP-BSIs treated with CAZ-AVI are Charlson comorbidity index ≥ 2, presence of septic shock, neutropenia, an INCREMENT score ≥ 8, lower respiratory tract infection and CAZ-AVI dose adjustment for renal function^7–9^. Prolonging CAZ-AVI infusions to ≥ 3 h have been shown to reduce mortality in one study^10^. In our cohort, according to Cox Regression analyses, age, severe neutropenia, and initiation of CAZ-AVI on the day of blood culture was obtained were independent clinical determinants of mortality.

A major strength of the study is to demonstrate the role of CAZ-AVI monotherapy in improving hospital survival in a country where OXA-48-producing *K. pneumoniae* is prevalent. In particular, early initiation of CAZ-AVI was shown to result in a significant increase in survival rates compared to switching to last-resort treatment. In addition, starting CAZ-AVI treatment on the day of blood culture collection was found to significantly reduce the 30-day risk of mortality. There are limited data on this topic and we believe that our study will provide valuable information on CAZ-AVI monotherapy for CRKP-BSIs in OXA-48 endemic areas. Another important strength of the trial is its multicenter design, which increases the generalizability of our findings.

However, our study has some limitations. Firstly, its retrospective nature and the use of real-life data limit the ability to control for certain factors, such as the inability to determine carbapenemase types. OXA-48 was first identified in Turkey in 2001 and since then has become the predominant carbapenemase, particularly in *Klebsiella pneumoniae* isolates^2,13^. The most frequently detected carbapenemase type was OXA-48 (74.5–96.6%), followed by NDM (3.2–19%) and KPC (< 5%) in many studies conducted in Turkey^14,15,18–20^. Although OXA-48 was identified as the predominant carbapenemase type in studies from that period and it was assumed that most strains were OXA-48 producers based on CAZ-AVI susceptibility, the lack of direct confirmation of OXA-48 production in all CRKP isolates limited our findings. Secondly, our study did not differentiate between primary, secondary or catheter-related BSIs. Since different sources of infection may be associated with varying prognoses and treatment responses, this information could influence the results.

Finally, its retrospective nature may lead to potential limitations, such as selection bias and unmeasured confounding factors.

## Conclusion

In conclusion, this multicenter retrospective cohort showed that the mortality was 33% and CAZ-AVI monotherapy is an important treatment option in CRKP-BSIs in OXA-48 endemic area. Additionally, our study also confirmed that early initiation of CAZ-AVI is more convenient than switching to CAZ-AVI from a last-resort regimen. In regions where OXA-48 is endemic, prospective studies including the molecular characterizations are needed.

## Data Availability

Data availability statement: The datasets used and analysed during the current study available from the corresponding author on reasonable request.

## Abbreviations

BSIs: Bloodstream Infections
CAZ-AVI: Ceftazidime-Avibactam
CI: Confidence Interval
CRKP-BSIs: Carbapenem-Resistant Klebsiella pneumoniae Bloodstream Infections
CRKP: Carbapenem-Resistant Klebsiella pneumoniae
CRE: Carbapenem-Resistant Enterobacterales
EUCAST: European Committee on Antimicrobial Susceptibility Testing
HR: Hazard Ratio
KPC: Klebsiella pneumoniae carbapenemase
MALDI-TOF: Matrix-Assisted Laser Desorption/Ionization-Time of Flight
NDM: New Delhi Metallo-beta-lactamase
OXA-48: Oxacillinase-48
SPSS: Statistical Package for the Social Sciences
USA: United States of America
